# A rare case of a male child with post-zygotic de novo mosaic variant c.538C > T in *MECP2* gene: a case report of Rett syndrome

**DOI:** 10.1186/s12883-021-02500-5

**Published:** 2021-12-02

**Authors:** Jhanvi Shah, Harsh Patel, Deepika Jain, Frenny Sheth, Harsh Sheth

**Affiliations:** 1 FRIGE’s Institute of Human Genetics, FRIGE House, Jodhpur Gam Road, Satellite, 380015 Ahmedabad, India; 2 Zydus Hospital, Ahmedabad, India; 3 Shishu Child Development and Early Intervention Centre, Ahmedabad, India

**Keywords:** Rett syndrome, Male, Post-zygotic, de novo variant, Mosaic, MECP2

## Abstract

**Background:**

Rett syndrome (RTT) is characterized by a normal perinatal period with a normal head size at birth followed by normal development for the first 6 months of life followed by gradual deceleration of head growth, loss of acquired purposeful hand skills, severe expressive and receptive language impairment, severe intellectual disability and gait and truncal apraxia/ ataxia. It is caused due to mutations in the *MECP2* gene and follows an X-linked dominant mode of inheritance. It was observed exclusively in females and was believed to be lethal in males. In contrast to this belief, several males were identified with RTT upon genetic analysis, however, most males expired by the age of 2 years due to neonatal encephalopathy. The ones that survived beyond the age of 2 years, were attributed to the presence of an extra X chromosome (co-occurrence of Klinefelter and RTT) or the ones having mosaic cell lines. Only 11 males with somatic mosaicism are known till date.

**Case presentation:**

This case reports an ultra-rare case of a male affected with RTT surviving beyond the age of 2 years due to post-zygotic de novo somatic mosaicism. He was identified with a known pathogenic variant c.538C > T (p.R180*), which to the best of our knowledge is exclusively seen in females and has never been reported in a male before.

**Conclusion:**

The present case is the first report of a mosaic male affected with RTT from India. The present report also carried out genotype-phenotype correlations across surviving mosaic males with RTT. We also postulate the effect of variant type, position along the gene and the variant allele fraction in different tissue types to be correlated with disease severity.

## Background

In 1966, Andreas Rett, a neurologist, described a syndrome namely “cerebral atrophy and hyperammonemia” which was observed to confine to girls; which eventually came to be known as Rett syndrome (RTT). This syndrome was clinically characterized by autistic behavior, gait apraxia, stereotyped hand movements and loss of facial expression, having an age of onset between 6 and 18 months [1]. By the time the underlying cause of RTT was identified, over a 1000 cases of girls showing the described phenotype were known. Most of the female probands had European ancestry and a prevalence of 1:10,000 to 1:15,000 girls was estimated [2]. Even in the absence of a cause at the molecular level, a diagnosis of RTT could be made by its striking phenotype and natural course as described in the consensus data [3]. The consensus described a necessary and supportive criterion for the diagnosis of RTT. The necessary criteria included a normal perinatal period with a normal head size at birth and normal development for the first 6 months of life, followed by gradual deceleration of head growth, loss of acquired purposeful hand skills, severe expressive and receptive language impairment, severe intellectual disability and gait and truncal apraxia/ ataxia. Whereas, breathing difficulties, seizures, spasticity, scoliosis and growth retardation were included in the supportive criteria. At this point, although no males were reported, the consensus took males into account as well [3]. Occurrence of RTT was by and large sporadic (95%) with a few familial cases being reported. Linkage analysis in familial cases led to the suggestion of Xq28 as the critical region associated with RTT [4]. The presence of asymptomatic carrier females was attributed to non-random X-skewed inactivation [5]. With no males reported with the same phenotype, an X-linked dominant inheritance with lethality in males was hypothesized [6]. The first male to be affected with RTT was identified in a familial case that further supported an X-linked gene as the cause of RTT [6]. Mutations in the *MECP2* gene were identified as the cause of RTT in 1999, three decades after its first clinical description [7]. The gene encodes methyl-CpG-binding protein 2 (MeCP2) that encompasses two critical domains, a highly conserved 85 amino acid long methyl-binding domain (MBD) and a 104 amino acid long transcription repression domain (TRD). Functionally, MeCP2 acts as a transcriptional repressor by selectively binding to the CpG dinucleotides in the genome [8, 9]. Point mutations (SNVs and indels), exon(s) deletion and *MECP2* gene duplication seen in male probands expanded the mutational spectrum of RTT. Eleven males have been reported worldwide till date with RTT as a result of somatic mosaicism [10–17]. An ultra-rare case of RTT in a male child with autism and an additional phenotype of polydactyly due to somatic mosaicism is presented here, adding to the limited repertoire of surviving males affected with RTT. This is the first case report of a male with RTT as a result of somatic mosaicism from India.

## Case presentation

**Fig. 1:**
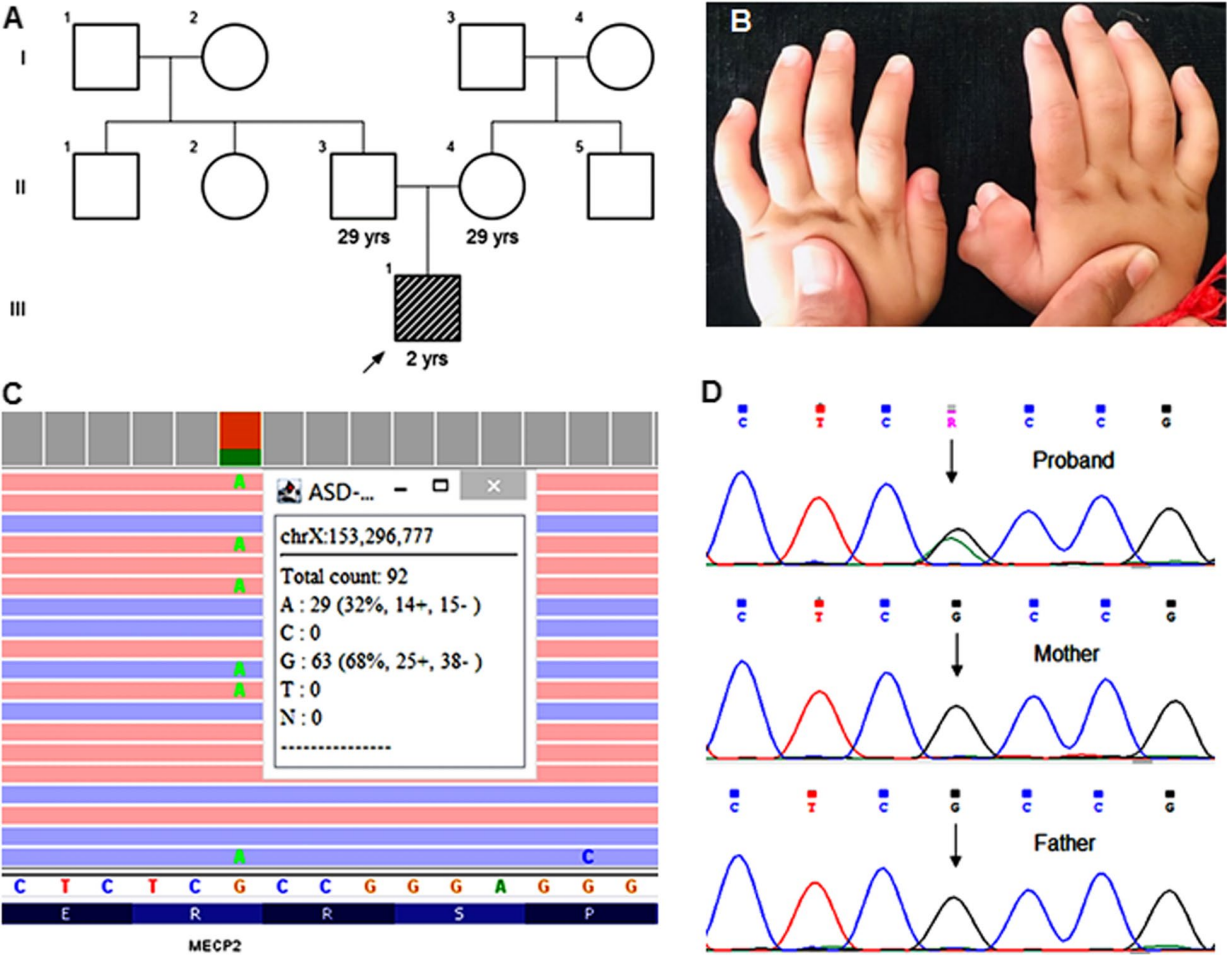
Pedigree chart of present case (1A). Affected individual is shaded in grey stripes. Unilateral right pre-axial polydactyly (1B). IGV depicts the causative variant in only 32% of the reads (1C) and Sanger sequencing chromatograms of the proband and his parents (1D)

A 2 years 7 months male proband was born to a non-consanguineous young couple (Fig. 1A). He was born prematurely at 32 weeks by cesarean section. He weighed 2.1 kg at birth and cried soon thereafter. He presented with apnea as a result of prematurity and was kept under observation in the NICU. Furthermore, he had a clinical history of global developmental delay and attained smiling, head holding, sitting and standing at 3, 7, 12 and 29 months respectively. He had difficulty standing for periods longer than ten minutes and currently he can only walk with support. He could only communicate via babbling. At 1 year 9 months’ age, he was admitted for an episode of febrile seizure and was started on an antiepileptic treatment soon thereafter. On examination, his head circumference, height and weight measured 48 cm, 87 cm and 12 kg respectively, all of which were normal for his age. Detailed clinical phenotyping suggested sudden tightening of the body, unilateral pre-axial polydactyly (Fig. 1B) and simian crease on the right hand along with autism spectrum disorder diagnosed according to DSM-5 criteria. His MRI of the brain was normal, whereas, EEG soon after the first episode of seizure revealed abnormal background activity with occasional right anterior epileptiform activity. Currently however, EEG suggests abnormal slow background for his age with occasional bihemispheric anterior dominant epileptiform activity.

Chromosomal microarray (CMA) and whole exome sequencing (WES) were performed on the genomic DNA extracted from peripheral blood of the proband to identify the genetic cause of his condition, subsequent to receiving institutional ethics committee approval as per the Helsinki declaration and a written consent from his guardians. No copy number variations i.e. deletions and/or duplications of pathogenic significance were detected using Affymetrix Cytoscan 750 k GeneChip (Affymetrix, USA). Thus, cytogenetic aberrations with resolution of 150 kb or greater were unlikely to be responsible for clinical features. Simultaneously, whole exome sequencing (WES) was performed. Agilent SureSelect v6 enrichment kit (Agilent, USA) was used for capturing of the exons and exon-intron boundaries. The library prepared was sequenced to mean > 80-100x coverage on Illumina HiSeq platform (Illumina, USA). Sequences obtained were aligned to the human reference genome (GRCh37/ hg19) using BWA [18]. SNVs and indels were called using GATK v4.1 [19]. Gene annotation was performed with JANNOVAR [20]. Phenotype driven variant filtration and prioritization was performed using Exomiser v12.1.0 [21] with available phenotype information translated in HPO terminologies. A likely heterozygous single base pair substitution in exon 3 of the *MECP2* gene c.538C > T (chrX:g.153296777G > A; Depth: 92x) that results in a stop codon and premature truncation of the protein at codon 180 (p.Arg180Ter; ENST00000453960.2) was detected in approximately 32% of the sequencing reads (92x) suggesting mosaicism (Fig. 1C). It is a known pathogenic, nonsense variant (SCV001447189.1). This variant has not been reported in the 1000Genomes [22] and gnomAD [23] databases. The observed variation lies in the Methyl-CpG binding domain of the MeCP2 protein and has previously been reported in multiple patients affected with Rett syndrome (SCV001447189.1). Validation of the variant and parental segregation analysis by Sanger sequencing showed the variant to be present in the child c.[538C=/538C > T] [p.R180=/R180*]. His parents were identified to carry the wildtype allele, confirming a post-zygotic de novo event in the proband (Fig. 1D). The variant was classified as pathogenic according to the ACMG-AMP classification system [24] and ClinGen framework [25] considering the following criteria: PVS1 (very strong), PP5 (very strong), PS3 (strong), PM2 (moderate), and PP3 (supporting).

To the best of our knowledge, this is the first time the aforementioned variant has been identified in a male, moreover in a mosaic state. Only 11 males with somatic mosaicism for mutations in the *MECP2* gene affected by RTT are known till date. The genotype and phenotype of all 12 males (including the child in the present study), showing somatic mosaicism were compared and have been described in Table. 1. The age at the time of evaluation ranged from 2 years 5 months to 14 years. All of these males had survived beyond the age of 2 years. Of these, most were diagnosed as classical RTT. Additional features included hypospadias and cryptorchidism that was seen in one child, otitis media and urinary tract infections seen in another case and polydactyly seen as an additional manifestation in the present case. Autism has been associated with RTT since the 1980s [1]. It was the first autism spectrum disorder (ASD) to have a genetic basis [7]. Autism was also a feature seen in the present case and has been reported in only one individual of the previously reported 11 cases, although, stereotypic hand movements which is a known phenotypic indication for autism was observed in most cases. Six individuals were found to have loss of function (LoF) variants and six had missense variants. Seizure phenotype was present in all cases that harboured LoF variants except in case 10 where the outcome of the variant affected amino acid residue 437. The observation of the absence seizures in case 10 could be due to the presence of the variant in a non-critical domain; an observation which is supported by differential effects of amino acid 270 and 273 in the MeCP2 protein, which are associated with causing neonatal encephalopathy with eventual death and survival with co-morbidities, respectively [26]. Probands harbouring missense variants were not detected with seizures, except in case 4.

**Table 1:** Genotype and phenotype of the present case and all reported males affected with Rett syndrome due to somatic mosaicism

		Case											
		1	2	3	4	5	6	7	8	9	10	11	12
**Reference**		Clayton-Smith et al. 2000	Armstrong et al. 2001	Topcu et al. 2002	Kleefstra et al. 2004	Psoni et al. 2010	Pieras et al. 2011	Zhang et al. 2018			Schönewolf-Greulich et al. 2018		Present case
**Age**		6y	14y	12y	11y	14y	4y	4y 4 m	2y 7 m	2y 5 m	8y	9y	2y 7 m
**Phenotype**		Classical	Classical	Classical	Classical	Classical	Atypical	Rett like	Classical	Classical	Classical	Classical	Classical
**Regression**		Yes	Unknown	Yes	Yes	Yes	No	No	Yes	Yes	Yes	Yes	No
**Main Criteria**	**Language & speech**	Present	Unknown	None	None	Poor	None	Poor	Lost	Lost	Lost	None	None
	**Hand skills**	Poor	Unknown	Lost	Lost	Lost	None	Poor	Lost	Lost	Poor	Poor	Poor
	**Stereotypic hand movements**	Present	Unknown	Present	Present	Present	Present	None	Present	Present	Present	Present	Present
	**Gait**	Ataxic	Unknown	None	Lost	Present	Ataxic	Poor	None	Poor	Ataxic	Poor	Poor
**Supportive Criteria**	**Breathing disturbance**	Present	Unknown	Absent	Unknown	Unknown	Absent	Unknown	Unknown	Unknown	Present	Present	Present
	**Bruxism**	Present	Unknown	Present	Present	Unknown	Present	Unknown	Unknown	Unknown	Present	Present	Unknown
	**Impaired sleep pattern**	Absent	Unknown	Unknown	Present	Unknown	Present	Unknown	Unknown	Unknown	Present	Present	Unknown
	**Abnormal muscle tone**	Present	Unknown	Present	Present	Present	Present	Unknown	Unknown	Unknown	Present	Present	Absent
	**Peripheral vasomotor disturbance**	Present	Unknown	Present	Unknown	Absent	Unknown	Unknown	Unknown	Unknown	Present	Absent	Unknown
	**Scoliosis/ kyphosis**	Present	Unknown	Present	Unknown	Absent	Unknown	Unknown	Unknown	Unknown	Absent	Present	Absent
	**Growth retardation**	Present	Unknown	Present	Unknown	Absent	Unknown	Unknown	Unknown	Unknown	Present	Present	Absent
	**Small cold hands and feet**	Present	Unknown	Present	Present	Absent	Unknown	Unknown	Unknown	Unknown	Present	Present	Unknown
	**Inappropriate laugh/ screening spells**	Unknown	Unknown	Present	Present	Absent	Present	Unknown	Unknown	Unknown	Present	Absent	Present
	**Diminished response to pain**	Unknown	Unknown	Unknown	Unknown	Unknown	Unknown	Unknown	Unknown	Unknown	Present	Present	Absent
	**Intense eye communication**	Unknown	Unknown	Unknown	Unknown	Absent	Present	Unknown	Unknown	Unknown	Absent	Absent	Unknown
**Seizure**		Present	Absent	Present	Present	Absent	Present	Absent	Absent	Absent	Absent	Present	Present
**Seizure onset age**		3y	NA	5y	2y 9 m	NA	2y	NA	NA	NA	NA	1y	1y 9 m
**Additional manifestations**		Nil	Nil	Hypospadias, cryptorchidism	Nil	Otitis media	Nil	Nil	Nil	Nil	Nil	Nil	Polydactyly
**Autism**		No	No	No	No	Yes	No	No	No	No	No	No	Yes
**Genotype**		c.241_242del (p.Gly81 Glnfs*9)	c.398G > A (p.Arg133His)	c.808C > T (p.Arg270*)	c.473C > T (p.Thr158Met)	c.316C > T (p.Arg106Trp)	c.360 T > G (p.Tyr120*)	c.317G > A (p.Arg106Gln)	c.316C > T (p.Arg106Trp)	c.353G > A (p.Gly118Val)	c.1308dup (p.Gln437Serfs*50)	c.808C > T (p.Arg270*)	c.538C > T (p.Arg180*)
**Percentage of variant allele fraction**		Unknown	Unknown	36%	~ 25%	Unknown	~ 10%	6.50%	26.32%	20.11%	9% (blood) and 24.8% (muscle biopsy)	37%	32%

## Discussion and conclusion

RTT was originally known to be restricted to females with a presumption of lethality in males. Only after the identification of a familial case of RTT in male, was the hypothesis of male lethality disregarded. Since then, many males affected with RTT have been reported in the literature with most of them expiring before the age of 2 years. Many revisions in the phenotype and genotype were made once the variants in the *MECP2* gene were identified as the cause of RTT. At present, RTT in females range from a classical RTT phenotype to a variant/ atypical RTT that could either be more severe or mild than the classical form and females with isolated learning difficulties. In contrast, the phenotypic spectrum in males range from the most common severe neonatal encephalopathy to pyramidal signs, parkinsonism, and macroorchidism (PPM-X) syndrome to severe syndromic/ nonsyndromic intellectual disability. In mosaic males, the phenotype has previously been suggested to bear a resemblance with females affected with RTT harbouring heterozygous mutations in *MECP2* gene. Interestingly, our case also presented with a unilateral pre-axial polydactyly, a phenotype which has not been observed in any other mosaic male RTT cases previously.

There are 4 potential scenarios where a male could have RTT: (1) sporadic cases due to germline de novo mutation; (2) males with Klinefelter syndrome; (3) males with mosaicism due to post-zygotic somatic event/s and; (4) familial cases with neonatal encephalopathy in males due to gonadal mosaicism in the mother. Most males with RTT expire by the age of 2 years. Surviving males with classical RTT have been attributed to somatic mosaicism or the presence of an extra copy of X chromosome [14]. The surviving male in the present case report was detected with a known pathogenic variant. This variant was first identified in the year of 1999, in multiple females [27]. However, to the best of our knowledge, no male has ever been reported with the same mutation. This variant has previously been observed exclusively in females with classical RTT. The presence of the same variant in the surviving male of the present case can be attributed to somatic mosaicism caused by a post zygotic event.

**Fig. 2:**
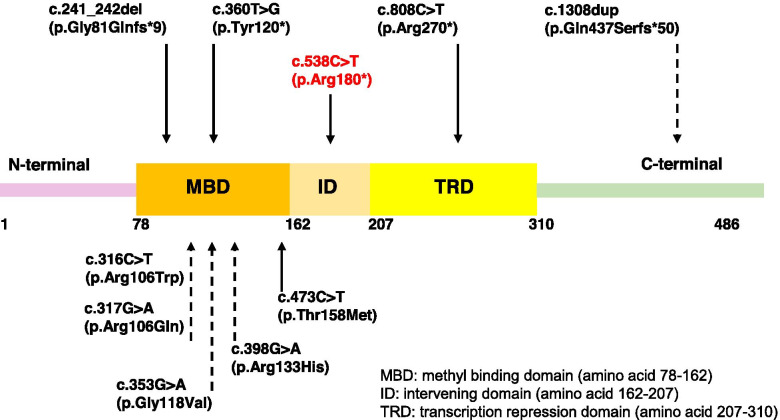
Schematic representation of MECP2 protein along with the corresponding domains and variants observed in mosaic males. Variant highlighted in red corresponds to the present case. Solid and broken arrows correspond to the variants associated with and without seizure phenotype, respectively

A prior study on the mutational spectrum in males with a germline mutation suggests that the location of the variant influences disease onset and severity [26]. Of the total 12 cases, all except one with the mutation affecting codon 437 and amino acids downstream to it, harboured mutations before or at amino acid 270 of the *MECP2* gene. LoF variants present before or at amino acid 270 were observed to be associated with seizures which was not observed in most cases with missense variants (*n* = 5 v/s 1; Table 1; Fig. 2). Based on this observation we hypothesize that not only the position but also the type of variant could contribute to the phenotype and severity of the disease in somatic mosaic males with RTT. Furthermore, the proportion of variant allele fraction in different cell lineages could also influence disease phenotype and severity. Mathematical modeling and simulation has shown mosaic variants to be shared among various tissues when mutant alleles were present in greater proportion [28, 29]. Whilst correlation between disease severity and variant allele fraction in the peripheral blood leukocyte has not been observed, assessment of variant allele fraction in other tissue types and correlation with phenotypic spectrum and severity in these patients remains to be explored. Comparison of the genotype and phenotype also suggests that despite mosaicism, most males have classical RTT phenotype. This case adds to the literature of surviving males with RTT attributable to post-zygotic de novo somatic mosaicism.

In conclusion, males with RTT have long since been observed once the initial hypothesis of male lethality was disregarded. However, most males with RTT were known to have severe neonatal encephalopathy and expire by the age of 2 years. RTT males seen surviving beyond the age of 2 years were attributed to presence of an extra X chromosome (Klinefelter syndrome – 47,XXY) or the presence of mosaic cell lines with a balanced chromosomal constitution. Herewith, we reported an ultra-rare case of male with RTT surviving beyond the age of 2 years. The present report also carried out genotype-phenotype correlations across surviving males with RTT. We also postulate the effect of variant type, position along the gene and the variant allele fraction in different tissue types to be correlated with disease severity.

## Data Availability

Data generated and analyzed during the study are included in the article.

## Abbreviations

RTT: Rett syndrome
MeCP2: Methyl-CpG-binding protein 2
MBD: Methyl-binding domain
TRD: Transcription repression domain
CMA: Chromosomal microarray
WES: Whole exome sequencing
ASD: Autism spectrum disorder
DSM-5: Diagnostic and Statistical Manual of Mental Disorders – 5
Lof: Loss of function
